# Integrated biophysical matching of bacterial nanocellulose coronary artery bypass grafts towards bioinspired artery typical functions

**DOI:** 10.1038/s41598-023-45451-2

**Published:** 2023-10-25

**Authors:** Jörn Hülsmann, Theresa Fraune, Baratha Dodawatta, Fabian Reuter, Martin Beutner, Viktoria Beck, Matthias Hackert-Oschätzchen, Claus Dieter Ohl, Katja Bettenbrock, Gabor Janiga, Jens Wippermann, Max Wacker

**Affiliations:** 1 Department for Cardiac Surgery, Medical Faculty, Otto von Guericke University, Magdeburg, Germany; 2 Laboratory of Fluid Dynamics and Technical Flows, Otto von Guericke University, Magdeburg, Germany; 3 Department Soft Matter, Otto von Guericke University, Magdeburg, Germany; 4 Chair of Manufacturing Technology with Focus Machining, Institute of Manufacturing Technology and Quality Management, Otto von Guericke University, Magdeburg, Germany; 5 Max Plank Institute for Dynamics of Complex Technical Systems, Magdeburg, Germany

**Keywords:** Translational research, Biomimetics

## Abstract

Revascularization via coronary artery bypass grafting (CABG) to treat cardiovascular disease is established as one of the most important lifesaving surgical techniques worldwide. But the shortage in functionally self-adaptive autologous arteries leads to circumstances where the clinical reality must deal with fighting pathologies coming from the mismatching biophysical functionality of more available venous grafts. Synthetic biomaterial-based CABG grafts did not make it to the market yet, what is mostly due to technical hurdles in matching biophysical properties to the complex demands of the CABG niche. But bacterial Nanocellulose (BNC) Hydrogels derived by growing biofilms hold a naturally integrative character in function-giving properties by its freedom in designing form and intrinsic fiber architecture. In this study we use this integral to combine impacts on the luminal fiber matrix, biomechanical properties and the reciprocal stimulation of microtopography and induced flow patterns, to investigate biomimetic and artificial designs on their bio-functional effects. Therefore, we produced tubular BNC-hydrogels at distinctive designs, characterized the structural and biomechanical properties and subjected them to in vitro endothelial colonization in bioreactor assisted perfusion cultivation. Results showed clearly improved functional properties and gave an indication of successfully realized stimulation by artery-typical helical flow patterns.

## Introduction

While cardiovascular diseases constantly lead the causes of death worldwide^1–3^, the prospect of survival after myocardial infarction (MI) improves by proceedings of the coronary artery bypass grafting (CABG) revascularization technique^1^. But CABG also installs an artificial biophysical niche by cross bridging the environments of the ascending aorta and the occluded coronary artery. After revascularization, this artificial niche needs to fulfill a cluster of native biophysical requirements which control the complex myocardial mass transport of nutrients and metabolites down to capillary flow and maintain its vascular biological functions. However, clinical reality proved that the only design to fulfill these demands sufficiently is derived by nature, more precisely the active biological adaption of autologous arteries like the internal thoracic artery (ITA)^1, 4^. Hence, no other graft, neither autologous veinous grafts like the great saphenous vein (GSV) nor any synthetic material overcomes functional impairments like competitive, low or turbulent flow^1, 5^, low shear stress^4, 5^ or overstressing vascular cells, which finally fosters pathological narrowing of the lumen by thrombosis, intimal hyperplasia or atherosclerosis^1, 5, 6^. In consequence, progress in the development of biomaterials allowing for biomimetic physiological performance of synthetic CABG grafts is highly called for and already created a wide variety of tools to defend causalities for the clinically observed pathologies^7–15^.

As the vascular biophysicalniche can be defined by reciprocally involved contact and flow derived stresses, like topography and shear stress, curvature and pressure or stiffness and wall strain^16^, tailoring biomimetic stimulation stresses can be realized by controlling mainly (i) elasticity and (ii) form &topography. On this matter, currently the generation of functional synthetic CAGB grafts still is blocked mainly by the unsolved challenge inmatching the biomechanical properties between CABG and the host in (i) and suffers from corresponding disturbances in flow and mechanotransduction^5, 17, 18^.

Interestingly, technical approaches in (ii) are seemingly much further progressed and provide a variety of structures to hamper the adhesion of thrombotic particles, prevent adverse flow events and guide endothelial colonization^7–15^. Consequently, integrated technologies in (i) and (ii) could greatly enhance proceedings towards clinical relevance^9, 19, 20^. At this level, biological hydrogels derived by growing bacterial nanocellulose (BNC) biofilms hold a naturally integrative character of both (i) and (ii) in its intrinsic architecture^21, 22^.This makes it a modular technology that elegantly combines all relevant scaffold properties. Furthermore, hydrogel scaffolds can be directly harvested from BNC biofilms, which already closely resemble the typical structure of Extracellular Matrix (ECM).Such BNC hydrogels already passed the classification as a safe biomaterial, which is accepted for clinical use^16, 23, 24^.

It is produced as a nonwoven matrix of a biofilm by cellulose producers as Komagateibacter *xylinus* at the air-liquid culture medium interface^23–25^. Kralisch et al. established a bioreactor-based production of medical-grade BNC directly from the bacterial culture broth at the headspace of the bioreactor^23, 25^. Today this process is employed to produce BNC patches holding medical approval e.g. for wound dressing (epicite® patch, JenaCell, Jena, Germany).

Basically, the same procedure, but utilizing form giving templates which are mechanically dipped into the culture broth which are then subjected to air incubation at the headspace, to allow the bacteria to transform the wetting into a BNC biofilm, is used to produce tubular BNC nonwovens as vascular scaffolds. This coating technology is called Mobile Matrix Reservor Technology (MMR-Tech) and was developed by Prof. Dieter Klemm (KKF Polymers, Jena, Germany)^19^.

Our earlier efforts in adapting MMR-Tech derived vascular BNC scaffolds for CABG grafting again proved biocompatibility, revealed good surgical handling and initially showed a multilayered ingrowth of host cells in a small animal model. But over a series of following large animal studies to assess modifications towards a smoother luminal surface, longer sized grafts continuously showed luminal narrowing^26–28^.

For further development, the MMR Tech bioreactor delivers mutable tools to set up the properties of the formed intrinsic BNC matrix of the biofilm. This holds a very essential difference to manually produced hydrogels, which properties can be defined directly by ingredients at their recipe^12, 29^. For BNC Hydrogels the recipe can only be adjusted indirectly by balancing variables and parameters of the bacterial productivity^21, 30, 31^. Consequently, in this study we want to use the freedom over (i) and (ii) as given by the integral nature of the growing biofilms, by combining the MMR-Tech tools with bioprocess engineering methodology to combine promising biophysical stimulation patterns into integrated multifunctional approaches for CABG prostheses.

Therefore, it appeared obvious to consider the known beneficial effects of mimicking artery typical axial oriented macro and microstructures which have been widely investigated and shown to direct endothelial cell growth^32–35^, to prevent cross flow and to provide a cleaning effect^36^, and further consider that for fiber-scaffolds, larger fibers and denser scaffolds better stimulated endothelial growth and physiology^37^.

Accordingly, our first motivation (**A**) was to find an integrated biomimetic design that can transfer a combination of these effects to the MMR-Tech technology, and which additionally provides sufficient artery-typical wall shear stress (WSS) stimulation under the artificial CAGB environment. Additionally, we tried to use findings from microfluidics, where angled micro topographies have been shown to induce helical flow patterns for micro-mixing applications^38^, to introduce helical flow patterns to BNC-CABG grafts via micro-topographic designs into our integrated approach. Inducing helical flow patterns is a relatively new motivation based on the growing evidence about the essential role of helical flow along arterial walls for maintaining its healthy physiology. Significance substantiates increasingly on its function to stabilize the mass transport, suppress adverse flow events, enhance oxygen supply to the wall, prevent the accumulation of low-density lipoprotein (LDL) particles and reduce the adhesion of blood cells^39^ in arteries of the systemic circulation as well as in coronary arteries^39–41^. At the same time spiral flow is under debate to improve the immediate clinical performance of CABG grafts by preventing flow disturbances after anastomosis, but technical realization remained rather elaborate and mostly relies on macroscopic shovels or swirling geometries^32, 33^.But next to this, the listed beneficial effects on vascular biology and the respectively expected impact on long-term patency, especially findings on reduced energy dissipation by aortic helical flow *in vivo*, potentially leading to optimal myocardial loading^39, 43^, drives our interest to find a suitable transfer to BNC-CABG technology. Here, a direct functional link to the biomechanical properties is given, so that both qualities may interact to either correct disturbances or eventually enhance their effects on damping remaining energy dissipation. Therefore, the introduction of helical flow while vectoring endothelial alignment for BNC hydrogel tubes that biomechanically matches to artery typical properties, represents our motivation (**B**) on finding an integrated artificial design that transforms the artificial CABG niche to the mentioned biofriendly functions. Therefore, we consider our motivation (B) rather as bioinspired than to be biomimetic.

## Results

### *Komagateibacter xylinus* cultivation

To produce hydrogels that combine increased load bearing properties and expose a dense and smooth fiber matrix at the luminal surface, which provides an increased volumetric-charge and attachment sites for endothelial colonization and further lacks potentially thrombogenic inhomogeneous rough areas, we increased the BNC density. Therefore, we replaced the previously used static K. *xylinus* pre-cultivations by dynamic bioreactor mass cultivation.

Here, the MMR Tech procedure requires conditions that inhibit BNC production in the culture broth but induce it only by air incubation. Consequently, enzymatic cellulose digestion and genetically engineered manipulations e.g. by inducible expression vectors, are not applicable. But the K. *xylinus* wildtype holds a natural regulation for both, cell growth and BNC productivity, just by oxygen availability^44^. This elegant effector can be easily controlled by standard pO_2_-control *(pO*_*2*_* = partial pressure of oxygen in the gas-phase*) strategies. Consequently, we evaluated the standard pO_2_ gas-mix control cultivation strategy at a setpoint of 4 vs. 10% pO_2_. First cultivations revealed high formation of BNC at all available surfaces in the bioreactor for the 10% setpoint, while decreasing it to 4 % resulted in largely suppressed BNC formation.

**Figure 1:**
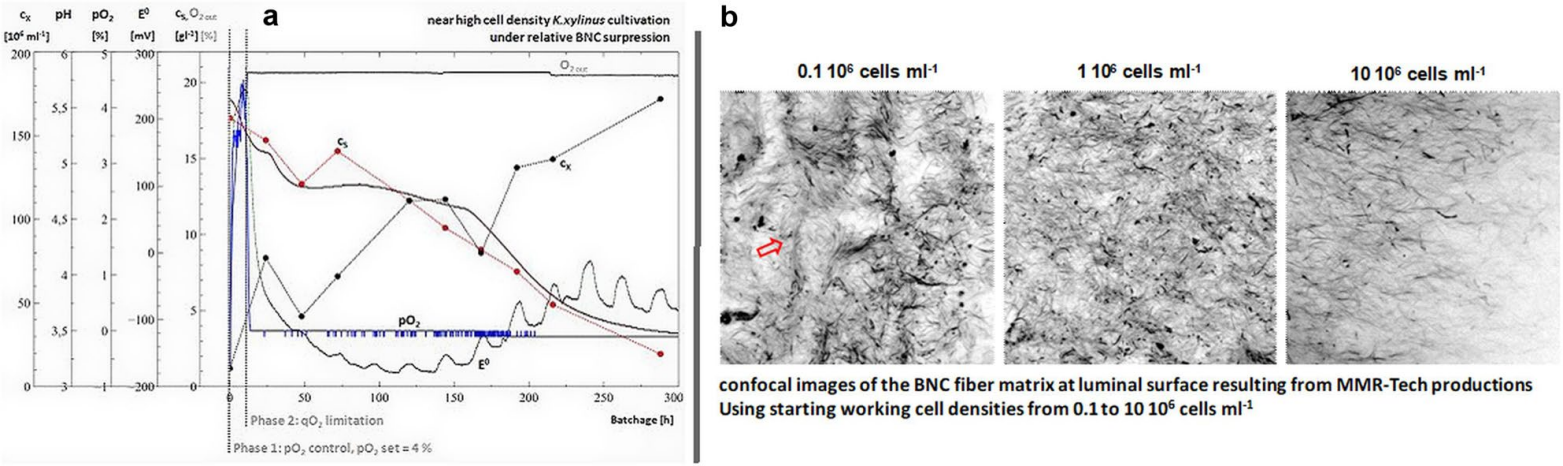
Komagataeibacter *xylinus* cultivation and luminal BNC fiber matrix after MMR Tech production of BNC hydrogel tubes. (**a**) process courses of pO_2_ controlled K. *xylinus* high cell density cultivation. c_X_ = cell density, c_S_ = glucose concentration, E^0^ = redox potential. The courses show the bacterial growth curve as c_X_ increases mostly linear under oxygen limitation. (**b**) Confocal images of the luminal BNC fiber matrix after staining with Calcofluor White. Images have the size of 170 × 170 µm.

In five cultivations, we reached an average of 102 ± 39 10^6^ cells ml^–1^ by a 20 ± 3.8-fold expansion in about 300 hours of processing. The process courses showed that after controlling the pO_2_ robustly at the setpoint for about 24 hours, it dropped down and remained at zero. Here, the range of oxygen feed into the manipulated gas-mix was maxed out and the process turned to stable oxygen limitation processing what then continued BNC suppression and growth-control via the gassing rate. The redox potential kept stable while pO_2_control but showed a parabolic pattern in the oxygen limitation phase, having its vertex at glucose concentrations below 15 gl^–1^. Subsequently it decreased again when c_S_ fell below 6 gl^–1^ and the pH reached values below 4 (Fig. 1a).

### Effecting the luminal surface

When using the produced bacteria to inoculate the MMR-Tech process, the working cell density c_X_W_ could still be increased by up to 2 powers of ten reaching about 10 × 10^6^ in contrast to 0.1 × 10^6^ cells ml^–1^, when compared to previous standard productions. Thereby produced BNC hydrogels then showed an evolution towards a smooth and dense fiber matrix at the luminal surface (Fig. 1b). Here, increasing c_X_w_ successfully could fill previously observed inhomogeneities and present a smooth luminal surface.

### Effecting the biomechanical properties

**Figure 2:**
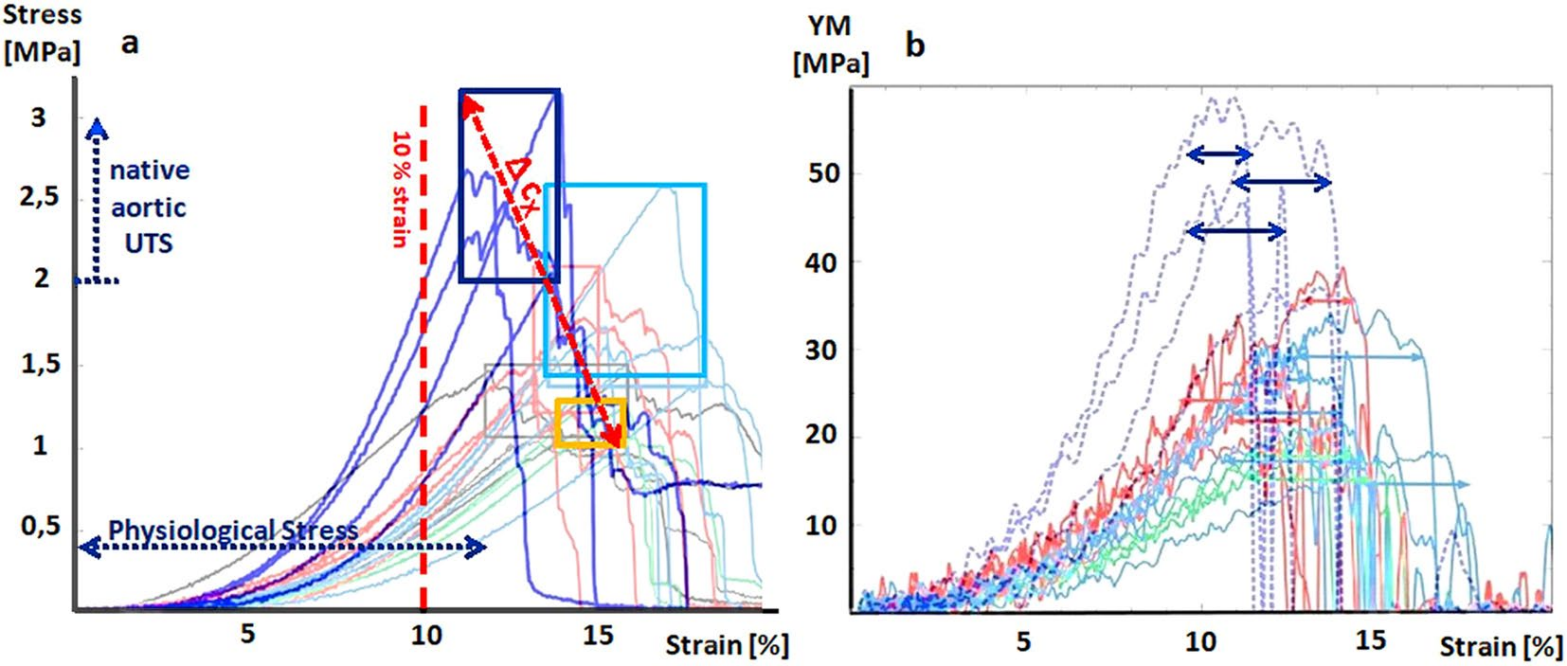
Biomechanical properties at varying working cell densities. (**a**) Overlay of stress strain relations of MMR-Tech derived BNC tube walls after uniaxial longitudinal tensile testing using a Tira-Test machine. Contrasted are tubes as produced at varying working cell densities c_X_w_ of K. *xylinus* ranging from 10^5^ (yellow) over 2 × 10^6^ (light blue) to 20 × 10^6^ (dark blue) cells ml^–1^). Respectively achieved ultimate tensile stress UTS and ultimate stains span the colored boxes. The red double arrow illustrates the shifting of these biomechanical properties by **∆c**_**X**_. Native-like aortic wall stress near physiological conditions and the range of maximal load is illustrated by the blue dashed lines. The graph gives an impression about how increasing c_X_may counteract overstretching BNC grafts at physiological loading and shows the increasing of the maximal strength towards native like values. (**b**) Overlay of the Youngs Modulus over strain derived by the middle difference quotient of the stress strain relations in (**a**). The horizontal double arrows span the linear hooke range, where the YM can be estimated to be constant.

Uniaxial tensile testing of BNC hydrogel tube walls, produced by increasing c_X_w_ under longitudinal stretching showed a shifting towards the typical J-formed Stress-Strain relation of collagenous soft tissues (Fig. 2). Apparently, for tubes of increased c_X_w_ the breakthrough to the hooke area of linear strain-resistance can be shifted towards the native-like 10 % mark (Fig. 2a,b) and ultimate tensile stress (UTS) could reach the native like aortic range of 2 to 4 MPa. Based on mean UTS of 2.55, 1.6 and 1.45 MPa and mean maximal strain of 12, 17 and 14 % respectively for c_X_w_ values of 10, 2 and 0.5 10^6^ cells ml^–1^, this shifting towards native like properties could be estimated to be effected by +0, 12 MPa stress (R^2^ = 0.9995) and – 0.35% strain (R^2^ = 0.5) per million K. *xylinus* cells in c_X_w_. This clearly shows that for physiological wall stresses, the risk of clinically problematic overdistensions leading to aneurism and thrombosis can be counteracted by increasing c_X_w_.

### Integration of functional luminal topographies

To realize the integrated biomimetic approach (**A**) of longitudinally arranged lamellar structures, we chose a regular groove and ridge pattern of 50µm in width and height. Here, the motivation was to try to combine the effects of cell-alignment in oriented clusters at this size^41^ and impacts on WSS stimulation. The respective form giving templates were prepared by 3D printing and subjected to MMR-Tech coating.

To realize the bioinspired approach (**B**) to combine a guided and vectored cell alignment with inducing helical flow along the wall, we prepared templates using a machine-grinding setup. Here, we aimed to arrange colonized cells to a locally predominantly longitudinally oriented arrangement in a rather acute angle that still can induce spiral flow patterns.

**Figure 3:**
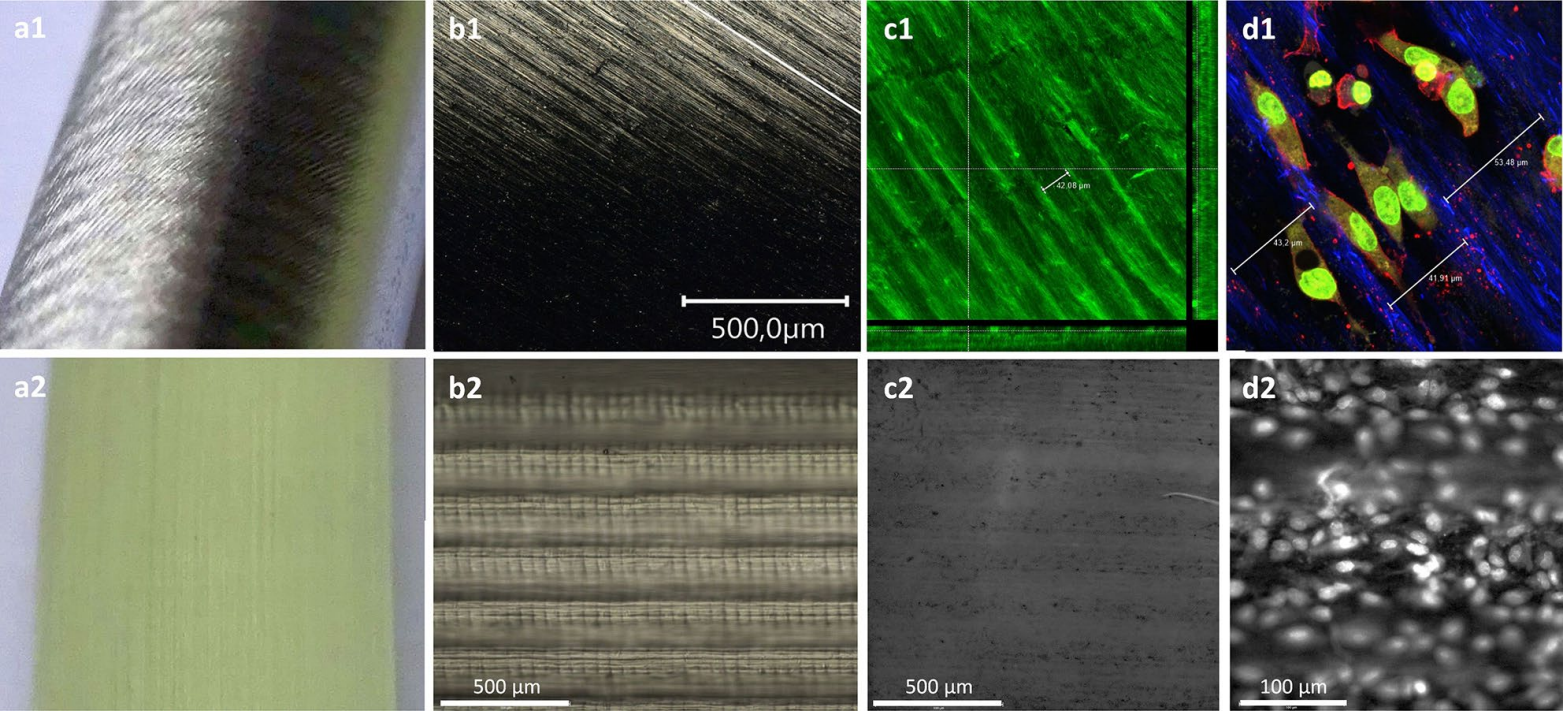
Template guided functional luminal micro-topography imprints. (**a1**,**2**) Cylindrical templates of 5 mm diameter for MMR-Tech coating. (**a1**) angled structures were manufactured by external longitudinal angled grinding. (**a2**) longitudinal intermediate range structures were prepared by 3D printing. (**b1,2**) microscopic images of the template surfaces. Apparently, both structures provide a lamellar structure in the intermediate range of 50 µm to 100 µm and sub groves in the micron range. (**c1,2**) microscopic images of the respective luminal BNC imprints after MMR-Tech coating. (**c1**) confocal image after Calcofluor White staining shows the angled lamellar profile providing sub grooves of about 40 µm width. (**c2**) fluorescence microscopic image after Calcofluor White stain shows the regular imprint of lamellar walls and groove cavities providing. (**d1,2**) Respective luminal micro topographies after in vitro colonization with endothelial cells (EC). (**d1**) EC’s accept the angled grooves and align according to the given orientation. (**d2**) EC´s accept and colonize both, lamellar walls and groove cavities and align according to the given orientation.

For both approaches, the manufacturing techniques produced the designed surface structures showing lamellar structures complemented by sub-grooves for helping cell arrangement (Fig. 3a1,2,b1,2). After subjection to MMR-Tech coating, the produced hydrogels gave clear imprints of the given topographies at the luminal surface (Fig. 3c1,2) which then could be accepted by endothelial cells after in vitro colonization (Fig. 3d1,2).

### In vitro performance

**Figure 4:**
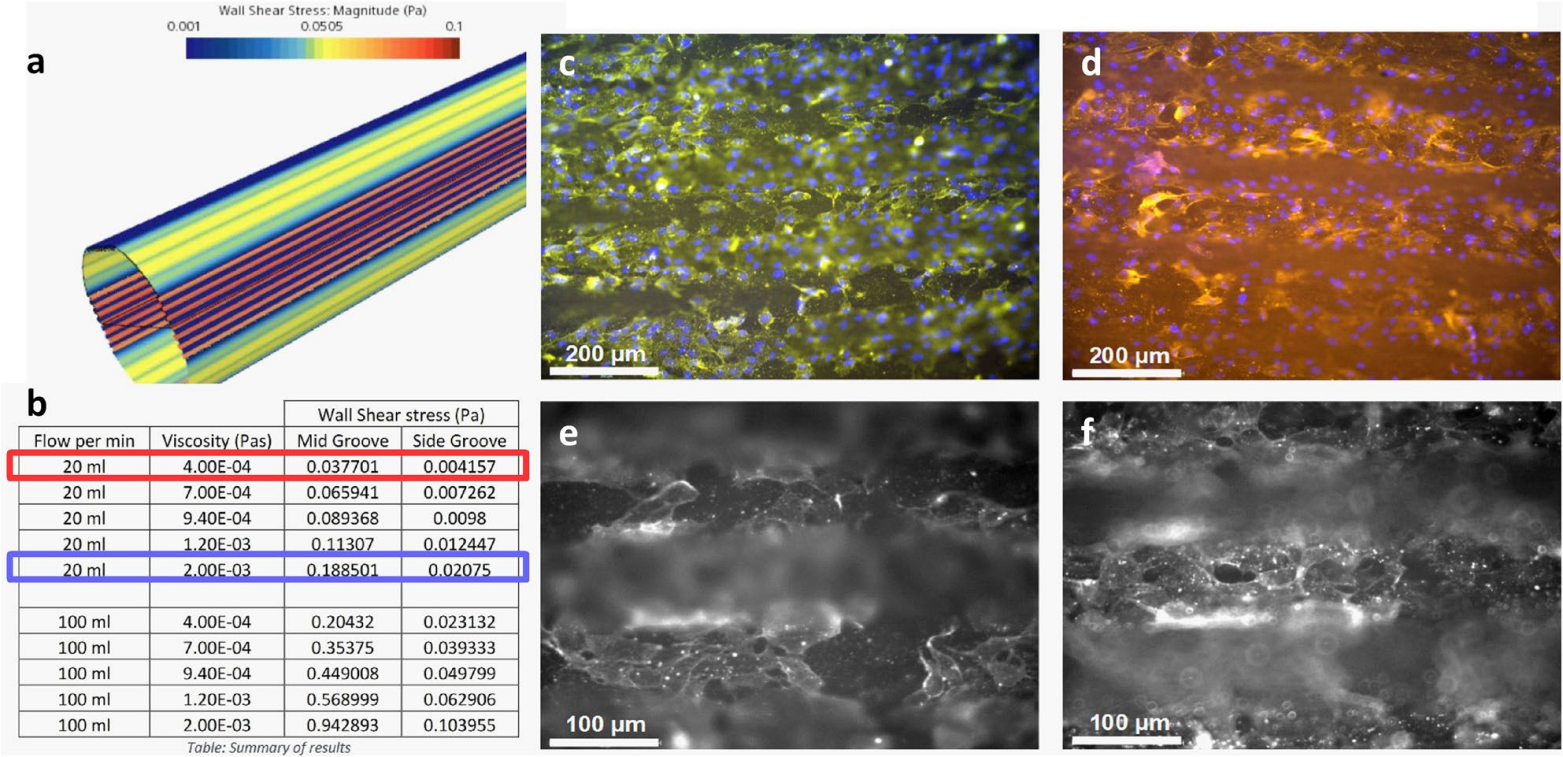
Success of in vitro colonization and bioreactor assisted perfusion for the integrated biomimetic approach: (**a**) Distribution of WSS stimulation for longitudinal microtopography. It reveals increased WSS at lamellar structures when compared to smooth surfaces. (**b**) Table listing resulting WSS at lamellar structures (Mid Groove) ad groove cavities (Side Groove) for increasing media viscosities from the given cell culture media (red box) to near blood viscosity (blue box). It can be seen that artery typical WSS of minimally 1 Pa cannot be achieved at low flow rates and cell culture media viscosity. (**c-f**) Colonized endothelial cells (EC) inside groove structures (**c**,**e**) and on lamellar walls (**d**,**f**) after F-actin (red in (**c**,**d**) and light in (**e**,**f**)) and DAPI (blue) staining. (**c**,**d**) EC´s densely colonize both, lamellar structures and groove cavities and align longitudinally according to the given topography. (**e**,**f**) F-Actin staining shows now pronounced cytoskeletal fiber arrangement and no differences in between lamellar walls and groove cavities.

Thereby produced BNC tubes, now providing a smooth and dense homogeneous luminal fiber matrix and either (**A**) biomimetic or (**B**) bioinspired functional topographies were evaluated on their effectivity by in vitro colonization of endothelial cells and short-term bioreactor assisted perfusion cultivation. Here, after discussion with local clinical perfusionists, the flow was set to 20 ml min^–1^ representing a low flow that is considered as still acceptable for clinical performance. The resulting flow patterns and the distribution of WSS stimulation were complemented by computational fluid dynamics (CFD). For the topography in (**A**) this proved 2-fold increased WSS at lamellar structures when compared to smooth surfaces, but decreased stimulation at the groove bottoms (Fig. 4a). It further showed that only 3% of an artery typical WSS stimulation of at least 1 Pa could be achieved for 20 ml min^–1^ using low viscous cell culture media of 0.4 mPas. This would be greatly increased for more viscous media. A viscosity of 2 mPas, so near to blood, would increase WSS to 20 % of native stimulation at 20 ml min^–1^ and almost to 100% at in vivo like flow rates of up to 100 ml min^–1^. For the groove bottoms all values are about 10-fold decreased (Fig. 4b).

However, after colonization and perfusion cultivation, endothelial cells accepted the given topography and densely colonized the luminal surface at both, lamellar walls and grooves. The cell bodies showed artery typical elongated shape and aligned in longitudinal orientation (Fig. 4c,d). But according the low WSS stimulation, we could not observe pronounced cytoskeletal fiber arrangements neither inside of the groove nor at lamellar walls (Fig. 4e,f)

**Figure 5:**
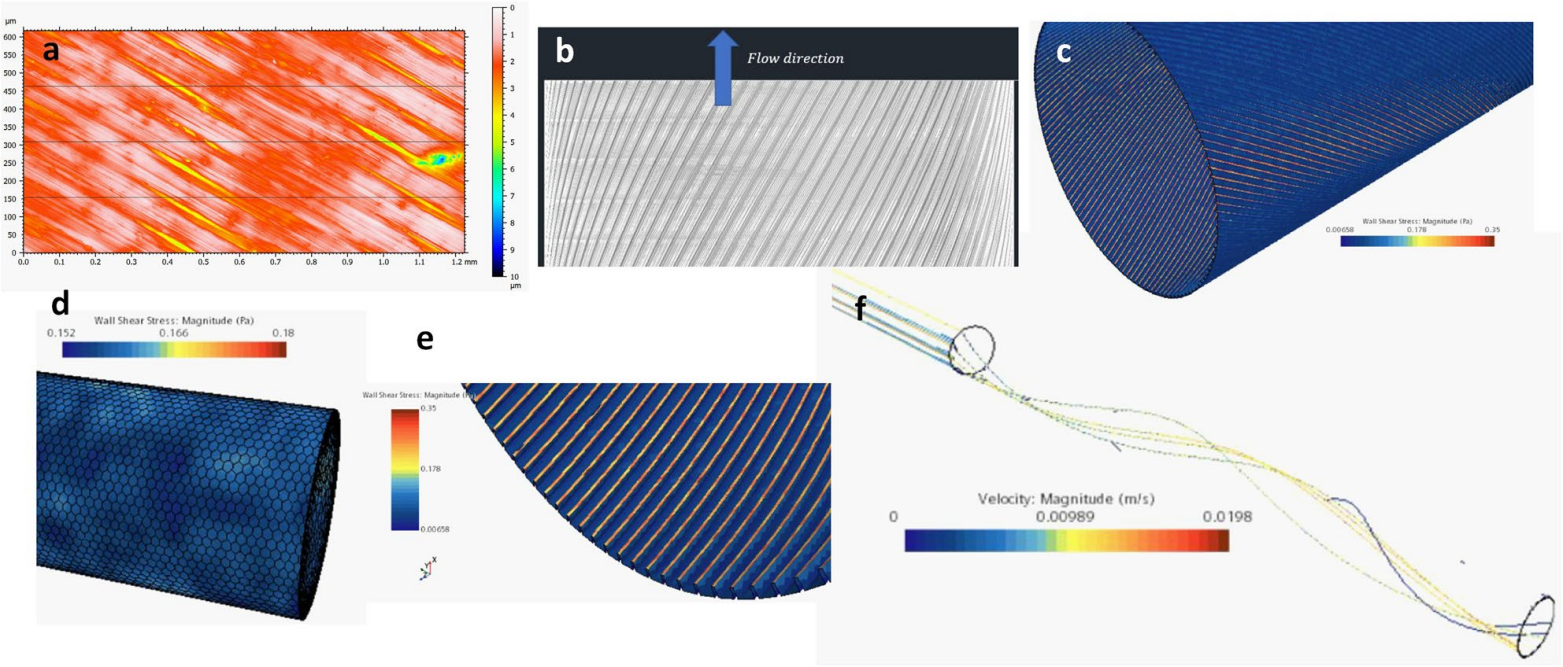
Geometry modelling and computational flow simulation for the angled micro topography. (**a**) Confocal image of the template surface after grinding. (**b**) Manually designed model to reflect the found topography in 3D. (**c**) Distribution of WSS under flow using the designed geometry in B. (**d**) WSS distribution for a smooth surface tube. (**e**) Distribution as shown in C at higher magnification to the luminal surface. (**f**) Resulting flow patterns at varying velocities showing helical flow along the luminal surface for the whole range of velocities.

For the topography in **B**, the resulting structures could not be directly transferred to CFD from a predetermined template design as it was given in A. Here, the luminal geometry needed to be manually approximated from optical surface measures of the produced templates and the confocal screens of the luminal BNC surface (Fig. 5a), which both are subject to measure uncertainties for exactly depicting the curved 3D architecture. For the approximation, as shown in Fig. 5b but neglecting sub grooves, the simulation showed a maximal WSS of 0.35 Pa at lamellar walls under in vivo flow conditions, again against a large drop for the groove bottoms (Fig. 5c–e). In contrast and similar to the geometry in A, for a smooth surface, the WSS stimulation would be restricted to about 50%. Nevertheless, the simulation clearly showed the presence of the targeted helical flow patterns along the tube wall for both, low in vitro and high in vivo like flow velocities (Fig. 5f)

**Figure 6:**
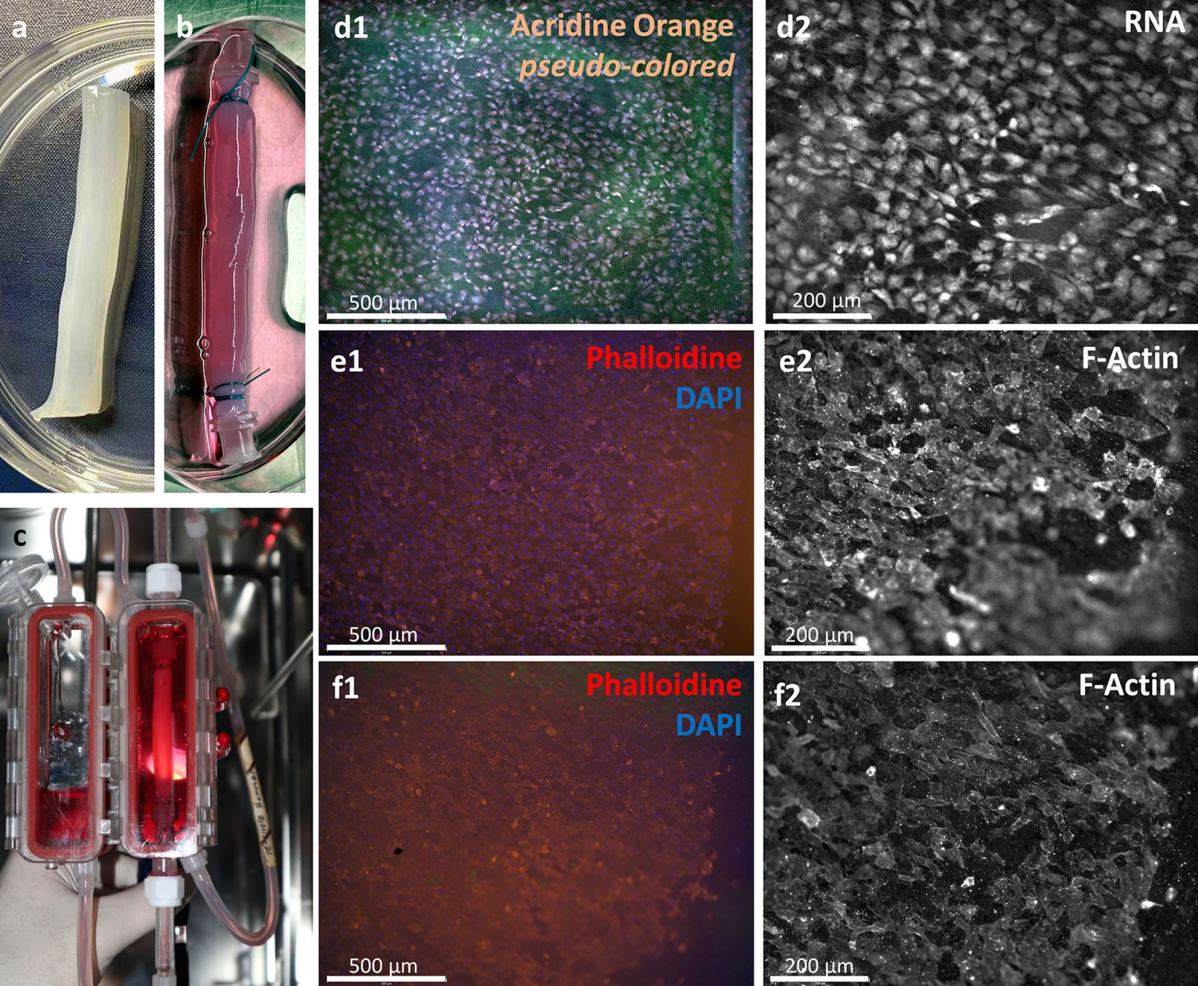
Success of in vitro colonization and bioreactor assisted perfusion for the integrated bioinspired approach: (**a**,**b**) produced BNC tubes before and after in vitro colonization. (**c**) colonized tube under perfusion in the bioreactor setup. (**d1**) Colonized endothelial cells (EC) on the luminal surface after Acridine Orange Staining for nuclei (blue = pseudo colored) and cytoplasm (red). EC’s densely colonize the whole surface and align according to the given angled topography. (**d2**) On higher magnification the cell bodies reveal mostly an elongated cell shape and show a vectored alignment following the given angle. (**e**,**f**,**1**) two more examples of densely colonized luminal walls after perfusion cultivation showing an equally distributed alignment over lamellar structures and sub grooves by interconnected cytoskeletal structures. (**e**,**f,2**) At higher magnification F-actin staining shows low pronounced fiber structures, indicating low WSS exposure.

After in vitro colonization and bioreactor assisted perfusion cultivation (Fig. 6a–c), endothelial cells accepted the angled luminal microstructures as well and aligned clearly according to the vectored topography. This divergence to the longitudinal central flow orientation underlines the actual presence of transversal helical flow along the tube wall. Colonized tubes showed mostly a dense endothelial coverage of the luminal surface at both, lamellar and groove structures (Fig. 6d–f1). However, in line with the observations for topography **A**, at low WSS stimulation in the in vitro setting, F-actin staining did not show pronounced cytoskeletal fiber arrangement and no local differences in it (Fig. 6e,f2).

**Figure 7:**
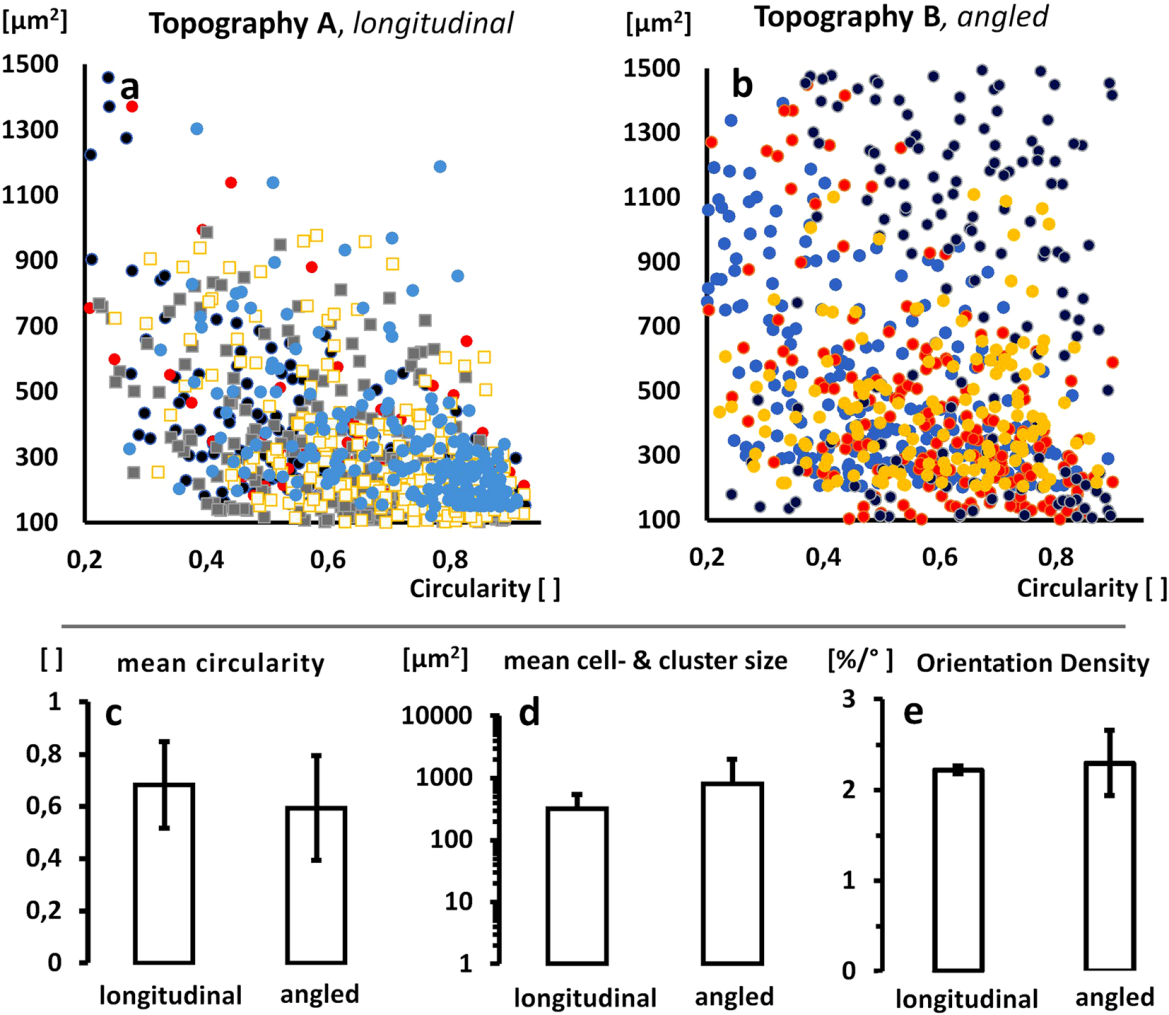
Vectorial character of endothelial colonization. (**a**,**b**) Distributions of vital cell and cell-cluster surface areas over its circularities for n=4 cultivations in A: Topography A (n=3 perfused: round marks, n=2 static: quadric marks) and B: Topography B (n=4 perfused: round marks). (**c**) Mean of found circularities for longitudinal (A) and angled (B) topographies. Apparently, the angled topographies stimulate a more pronounced artery typical ellipsoid shaping in contrast to the longitudinal structures in A. (**d**) Mean of found cell and cell-cluster sizes in square micrometers Here, the angled topography B shows much larger aligned cell-clusters, but at a high variance. (**e**) Orientation Density of cells colonized in the endothelial lining of topography A vs.B shows clearly impact to an oriented alignment.

By further quantification of the vectored impact on cell and cell-cluster growth, we found a clear effect on longitudinal shaping to by a mean circularity of 0.69 ± 0.17 and 0.69 ± 0.14 under perfused vs. static cultivation of topography **A**, but more distinct at 0.59 ± 0.2 for topography **B**, both representing clearly ellipsoid shape. The distribution of cell & cell-cluster surfaces over the circularity further show a more pronounced longitudinal shaping for growing clusters at larger interconnected endothelial linings, indicating the presence of vectored alignment for both topographies. However, for the angled topography **B**, this effect is apparently more pronounced. The higher share of larger and more shaped linings again underlines the presence of angled or transversal luminal flow, longitudinally oriented to the lamellar structures in topography **B**. In contrast, for static cultivations in topography **A**, the distribution appears more concentrated at a higher circularity and lower surface areas (Fig. 7a–c). However, due to rather high variances in the average cluster sizes of 323.6 ± 216 µm (perfused) and 323.6 ± 216 µm (static) in **A** vs. 804.8 ± 1176 µm under perfusion in **B**, we cannot determine a statistically significant difference in between the tested topographies (Fig. 7d). Finally, the orientation density (O.D.), which describes the share of cells which are aligned per degree of orientation angles, spanned by the bulk fraction of clearly oriented cells., shows a clear and roughly contrastable impact to the formation of an oriented organization of the endothelial lining by both topographies, **A** and **B**. Here the O.D. was calculated to be 2.22 ± 0.04 vs. 2.29 ± 0.36 % per degree for topography **A** vs. **B** (Fig. 7e).

## Discussion

Our motivation on BNC-CAGB prosthesis optimization by integrated approaches on guiding endothelial alignment and flow patterns could be successfully realized by using the MMR-Tech technology and thereby effecting the BNC density via the bacterial working cell density on the one hand and via vectored luminal micro topographies on the other hand. Doing so, at first, we addressed the long-lasting and widely investigated roadblock of biomechanical mismatching in between synthetic vascular grafts and the host^5, 17, 18^. In contrast to the MMR-Tech derived BNC-CAGB prosthesis that we used in earlier studies^26–28, 45^, we could increase c_X_w_ by two orders of magnitude and advance the biomechanical properties towards artery typical stress-strain relations. To better comply to the patterns of native arteries under uniaxial longitudinal tensile testing, the breakthrough of the tensile stress to a linear strain-resistance, the hooke area, should be moved below the 10 % strain mark and the ultimate tensile stress UTS should be moved to typical aortic UTS at 2 to 4 MPa^46, 47^. Both could be achieved simply by increasing c_X_w_. Thereby the UTS could be doubled, and the resulting strain could be reduced by roughly 40%, the latter already for physiological aortic wall stresses of below 0.5 MPa, what directly translates to a significantly reduced risk of forming aneurism and luminal narrowing by thrombosis.

Interestingly, though widely approached, the problem of mechanical mismatching for vascular grafts continues even to advanced approaches that made it to pre-clinical models. To estimate the potential clinical performance, these recently got contrasted to GSV^17^. One step beyond, a very recent study on BNC-CABG grafts used a surgical mesh to reinforce the mechanical properties and showed decreased luminal narrowing in vivo^48^. In direct comparison to the reported mechanical strength, increasing c_X_w_ in MMR-Tech production still resulted in about 7-fold higher total strength by withstanding a force of about 20 vs. 2.85 Newton.

At the same time, the luminal surface could be packed more tightly to eliminate inhomogeneity and provide densely distributed binding sites for endothelial colonization. Taken into consideration that by previous in vitro studies of our group, reducing the surface luminal roughness by using glass templates in the standard MMR-Tech process, utilizing classical microbial inoculation and static cultivation techniques, significantly improved the hemocompatibility by a reduced leukocyte interaction^28^, a further improvement for tubes derived by increased c_X_w_ may be predicted.

Additionally, we found that our fundamental preparative work on high cell density K. *xylinus* cultivation strategies, which we started and proceeded in 2020, also crosses very recent and meaningful industrial progress. In this context, Zywicka et al. 2021 presented a strategy to prepare high cell density K. *xylinus* culture broth for standardized inoculation of BNC producing processes under constant gassing and magnetic field stimulation in repeated fed batch airlift cultivation^49^. There, the authors report a drop of the dissolved oxygen to a final value of 2–3%. On the other hand, our initially pO_2_-controlled cultivations fell into a robust oxygen limitation at a pO_2_ of zero % after the setpoint of 4% was run down by the specific bacterial uptake. This fundamentally allows a robust growth control by the gassing rate under BNC suppression. Moreover, in contrast to the increasing redox potential under magnetic field stimulation as shown by Zywicka, for our process, the redox potential roughly followed previously observed conditioning patterns of the media and thereby may reflect process phases for optimal BNC productivity. This paves the way for on-line monitoring of process-phases and event-based process control in the future. Seemingly, the study by Zywicka represents the first published work on the optimization of K. *xylinus* inoculum production. Therefore, we are confident that our work can contribute scientific value on this aspect.

Regarding our intention to incorporate reciprocal contact and flow stimulation to the biological environment by vectored luminal topographies, we could transfer the imprinting method that was previously described as *guided assembly based biolithography*^50^ to the MMR-Tech methodology.

Thereby, for the integrated biomimetic approach in **A**, we could transfer the effect to align cells in clusters to a native like shape and orientation in longitudinal lamellar structures of intermediate range (50 µm) as previously reported^41^ and integrate regions of increased WSS exposure, targeting to induce artery-typical levels of shear induced anti-thrombotic signaling like e.g. NO production, what also is considered as an one important factor for the clinical success of ITA in contrast to GSV^1–3^. But at the same time, the groove cavities showed to reduce flow and WSS stimulation, so our initial design should be further optimized.

Balancing of biological effects in future studies will require to use viscous media and comprehensive bioanalytical evaluations. Given the artificial nature of the CABG environment it may be useful to deviate from pure biomimicry and prioritize on significant impacts to clinical functions. Artificial topography designs with distributed but high NO production may be feasible when adverse flow advents can be prevented at the same time.

Following this idea generally in our bioinspired approach in **B**, the angled topography showed indication to be effective on inducing artery typical helical flow patterns while also guiding a native-like cell shape and arrangement in endothelial colonization. Though the chosen geometry reveals even higher local WSS stimulation at lamellar structures than the longitudinal structures in A, it also shows a large drop for the grooves and therefore should be subjected to comprehensive further optimization as well. Nevertheless, after perfusion cultivation, colonized groove structures still showed a pronounced longitudinal shape of endothelial cells and a vectored alignment to large scale interconnected cell clusters. Interestingly, when contrasted to cardiac cells which populated the spatially highly organized myocardial extracellular matrix, the OD´s in both topographies, **A** and **B** show an increase of about 50 % in the resulting orientation density after colonization^51, 52^.

Of course, as this study mainly investigates the integrated impact of the biophysical properties of a scaffold, the presented functional evaluations still hold several limitations, especially regarding the translation towards its clinical relevance. In this context, we used a high cell density seeding method to observe the effects of the topography to endothelial alignment and induced flow in short model cultivations. Therefore, the simulation of in vivo like dynamics of endothelial growth is excluded from the study. But as the aspect of attracting cells in the clinical environment is investigated in parallel research of our group by post production modifications^53, 54^, these approaches can be combined in following studies. Further, the low wall shear stress stimulation by the low media viscosity and low flow rates blocks in depth investigations of cellular adaption towards an atherogenic niche. This is warranted to be investigated in comprehensive future studies as well.

## Methods

### *Komagateibacter xylinus* cultivation

BNC producing Komagateibacter *xylinus* bacteria were pre-cultivated using standard microbiological techniques. Therefore, colonies were grown on Hestrin Schramm (HS) agar plates and subsequently subjected to a series of pre-cultivations in test tubes and Erlenmeyer flasks before banking them in 50% glycerol on – 80 °C for further inoculations (standard aliquots = std-aq). For standard MMR-Tech BNC inoculum preparation, aliquots then were given to 500 ml Hestrin Schramm media in an Erlenmeyer flask.

Dynamic bioreactor assisted mass cultivation was realized using an Infors HT Multifors (Infors HT, Bases, Switzerland) bioreactor system providing two parallel glass vessels of 1 liter working volume. The system was equipped by blade stirrers (removed after cultivation 1) rings sparger for gassing, in-line sensors for temperature, pO_2_ –, pH -, and redox-potential, BlueSens off gas sensors for CO_2_ and O_2_ (Blue Sens Gas Sensor GmbH, Herten, Germany) and a clamp on heating jacket.

Before inoculation by adding 2 to 4 of the banked std-aliquots to 1 liter HS media, the pO_2_ value was decreased to zero by nitrogen gassing. After inoculation, pO_2_-gasmix control was started at a setpoint of 4% and a fix gassing rate. Cultivations were sampled daily and stopped after about 300 hours.

For harvesting, the culture broth was centrifuged at 2800 rpm and 4 °C using 50 ml falcon tubes. Cell pellets were pooled and re-suspended in 50 ml of 50 % glycerol HS media. 5 ml each were frozen at – 80 °C for usage as high cell density aliquots (hc-aq).

Thereby, we ran a series of 6 cultivations under full analytical monitoring.

### MMR-Tech BNC hydrogel tube production

Tubular BNC hydrogels were produced using one unit of the MMR-Tech bioreactor system (KKF Polymers, Jena, Germany). Inoculations were prepared by 500 ml of HS media and either a varying number of std-aliquots for standard productions, or hc-aliquots for productions at high working cell densities c_X_w_. Thereby, for each production process a batch 5 liters of HS media was inoculated and given to the bioreactor. The bioreactor system runs a series of mechanically dipping the installed arrangement of form giving templates to the culture broth and incubating the wetted templates against air. Induced by the high oxygen availability, K. *xylinus* bacteria then transform the fluid film of culture broth into a BNC stabilized biofilm. For standard productions, bamboo rods of 5 mm diameter were used as templates. Productions for tubes of type A (providing longitudinal micro topography) were equipped by self-prepared templates of medical grade 3D printing filament and by stainless-steel rods for type B (angled micro topography).

### Tensile testing

For uniaxial tensile testing of BNC tubes, a Tira Test machine was equipped with a 50 Newton force sensor. Tubes of approximately 5 cm length were longitudinally cut open and mounted to the grips by using grained paper strips to avoid slipping. Tensile testing was performed at a speed of 1 mm per second. The geometry of the samples was measured using a scale and used for normalization of distension to strain and measured force to cross-sectional wall stress. Thereby, we analyzed n=5 samples for standard productions under an n=3 for different working cell densities c_X_w_For all test, critical points at ultimate tensile stress UTS and ultimate strain US werepaired to the respective c_X_W_ and used for correlations using excel.

### Manufacturing of surface structured templates

Templates for productions of type A tubes were prepared by 3D printing using a Formlabs medical grade printer and belonging surgical guide resin (Formlabs, Somerville, MA, USA). Therefore, an axially oriented regular groove and ridge pattern design of 50 vs. 50 µm in width and depth was given to a rounded square rod design of about 16 cm length.

Templates for production of type B tubes: Stainless steel cylinders of 5 mm diameter and 19 cm length were machine grinded using a self-established setup for micro-shaping. Therefore, the cylinders were axially mounted into a rotatable stand and grinded by fixed movable Dremel in a 60° angle using grain size 60.

### HUVEC cell culture

Commercially available primary human umbilical vein endothelial cells (HUVEC) (ATCC PCS 100 series, ATCC Manassas, Virginia, USA) were expanded by standard cell culture techniques using Endothelial Cell Growth Media 2 (Promocell)supplemented by endothelial supplement mix, 10% fetal bovine serum and antibiotics. Before reaching a confluency level of 80%, cells were harvested using 1x trypsin-EDTA mix for 3 min at 37 °C and adjusted to density of 1 × 10^6^ cell ml^–1^ ingrowth media for the seeding suspension.

### Cell seeding and in vitro cultivation

BNC tubes at a length of about 6 cm were used for in vitro colonization. For mounting to the given connectors inside of the perfusion chamber of the bioreactor, male luer-lock connectors were tied to both ends using surgical suture. Tubes were then filled by the seeding suspension and subjected to 6 hours of incubation under stepwise manual axial rotation of 45° in intervals of 45 minutes, at 37 °C and 5% CO_2_. This procedure was repeated the next day, before colonized cells were allowed to adhere under static incubation over weekend. Subsequently, the colonized tubes were mounted to a specialized perfusion bioreactor setup (3D Cell Culture Pro, TA Instruments) installed inside of a Heracell 240i Cell culture incubator (Thermofischer) and run at 20 ml min^–1^perfusion flow at 37 °C and 5% CO_2_. Perfusion was realized using a Reglo ICC peristaltic pump (Ismatec, Grevenbroich, Germany) installed outside of the incubator. Perfusion cultivation then was performed for 4 days.

### Fluorescent staining and microscopy

After stopping cultivation, tubes were washed in cold phosphate buffered saline and fixed in 4% buffered paraformaldehyde and 10% sucrose for 10 minutes. Fluorescent staining of nuclei and cell bodies was realized using combined DNA and RNA staining by acridine orange (AO) according to the manufacture’s recommendations (cat.nr.:10127-02-3, Thermofischer). Fluorescent staining of the cytoskeleton was performed using phalloidin rhodamine (abcam), specifically interacting with F-Actin according to the manufacturer’s recommendations. Microscopic evaluation was done using an Evos FL Cell Imaging System (Thermofischer).

### CFD simulations

3D models of BNC tubes in Type A and B were designed using the commercial software AutoCAD and imported to STARCCM+ (CFD software from Siemens PLM) to conduct the simulations. Then STARCCM+ was used to generate the mesh, solve, undertake post processing and obtain the results. Based on the low Reynolds number, a laminar flow model was selected with steady state flow conditions to perform all the simulations. Special attention was given to estimate the respective distributions of WSS and flow velocities under perfusion at 20 vs 100 ml min^–1^.

### Image processing

The shaping of single cells and cell clusters was evaluated using ImageJ particle analysis of stained cell bodies at microscopic images. Therefore, first scale bars were used for calibration and images were prepared by background subtraction and thresholding. Thereby, resulting particles represented single cells or aligned clusters. Circularity, surface area and overall orientation angle of particles were analyzed and used for the evaluation of the distributions.

The orientation density (O.D.) was calculated as previously described^50, 51^. Briefly, the orientation angle of shaped cells was used to sort the digitalized cells and estimate the fraction of clearly oriented cells (FO). This fraction was divided by its angle range to estimate the O.D. to indicate the share of cells per degree of orientation that is trusted to represent the bulk orientation.

We analyzed a n = 3 respectively for colonized and perfused BNC tubes of Type A and an n = 4 for tubes of Type B. Thereof, two tubes were colonized by only one repetition of cell seeding and perfused by 10 ml min^–1^.

### Ethics declaration

No primary human or animal material was used. The used cell line was commercially acquired.

## Data Availability

The datasets used and/or analyzed during the current study available from the corresponding author on reasonable request.
